# Evolution, folding, and design of TIM barrels and related proteins

**DOI:** 10.1016/j.sbi.2020.12.007

**Published:** 2021-06

**Authors:** Sergio Romero-Romero, Sina Kordes, Florian Michel, Birte Höcker

**Affiliations:** Department of Biochemistry, University of Bayreuth, 95447 Bayreuth, Germany

## Abstract

Proteins are chief actors in life that perform a myriad of exquisite functions. This diversity has been enabled through the evolution and diversification of protein folds. Analysis of sequences and structures strongly suggest that numerous protein pieces have been reused as building blocks and propagated to many modern folds. This information can be traced to understand how the protein world has diversified. In this review, we discuss the latest advances in the analysis of protein evolutionary units, and we use as a model system one of the most abundant and versatile topologies, the TIM-barrel fold, to highlight the existing common principles that interconnect protein evolution, structure, folding, function, and design.

## Highlights

• In-depth sequence analysis reveals that the protein fold universe is more evolutionarily connected than previously assumed.
• Short ancestral fragments are observed to have propagated to many modern proteins and hint at possible evolutionary pathways.
• Experimental reconstruction of such events by chimeragenesis and directed evolution allows to test evolutionary relationships.
• Detailed knowledge of folding landscapes helps to understand evolutionary history and improve protein engineering.
• The ubiquitous and versatile TIM-barrel fold is a model system to explore evolution, folding, and design.

**Current Opinion in Structural Biology** 2021, **68**:94–104

This review comes from a themed issue on **Sequences and topology**

Edited by **Nir Ben-Tal** and **Andrei N Lupas**

For a complete overview see the Issue and the Editorial

Available online 13th January 2021

**https://doi.org/10.1016/j.sbi.2020.12.007**

0959-440X/© 2020 The Authors. Published by Elsevier Ltd. This is an open access article under the CC BY license (http://creativecommons.org/licenses/by/4.0/).

## Introduction

Structural and functional diversity in modern proteins is the result of diversification and optimization processes over the course of evolution. Studying these processes is useful to evaluate how different molecular mechanisms, like duplication and recombination, shape biophysical properties in proteins. Sequence and structural analysis suggest that numerous protein pieces, considered as evolutionary units, have been reused and combined to create higher complexity. In this context, what are the reasons for the recurring success of some of these units? What is their role in protein fold diversification? And how can we use the accumulated information to further our protein design goals?

**Figure 1:**
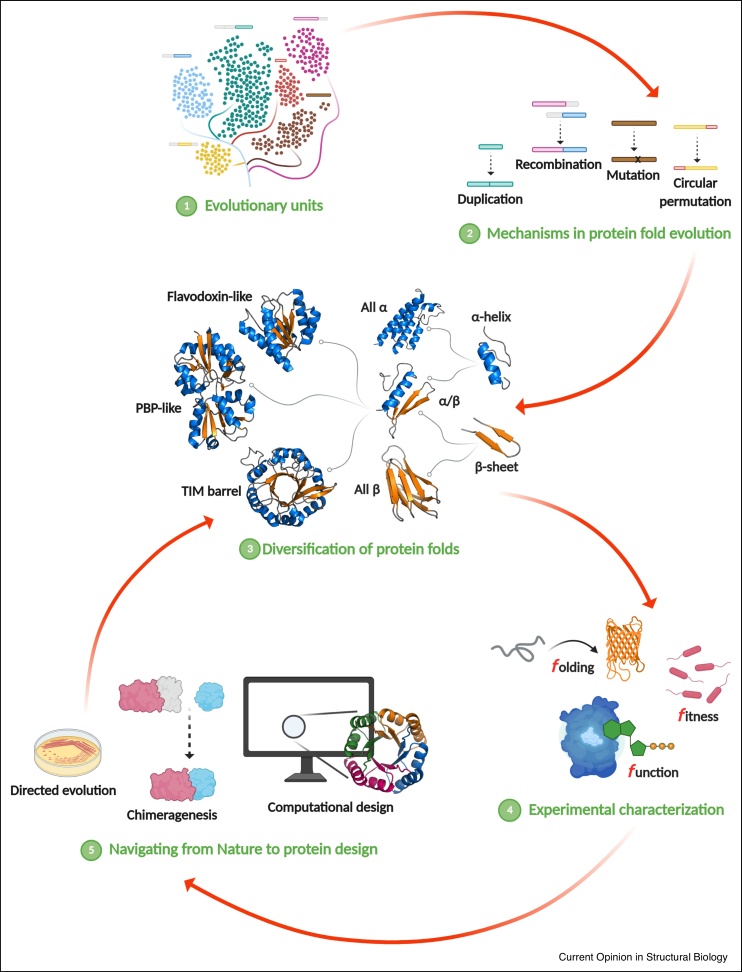
Schematic overview of the relationships between protein fold evolution, experimental characterization, and design approaches discussed in this review. The upper part of the figure shows how evolutionary units are reused through different molecular mechanisms to diversify protein folds. Experimental reconstruction of different evolutionary pathways and the analysis of folding, function, and fitness determinants in evolution increase our knowledge of the protein-based world and allow navigating from Nature to protein design as shown in the bottom part.

In this review, we try to unravel these mysteries by integrating different perspectives and approaches (Figure 1). We first discuss the current views of evolutionary units (Section ‘Current views of evolutionary units’). Then, we use the TIM-barrel fold as model system to analyze how our knowledge of the protein-based world is enhanced by the integration of evolutionary analysis (Section ‘Evolutionary events: fragments and natural TIM-barrel proteins’), experimental recreation of evolutionary events (Section ‘Recreating evolutionary events in the lab: chimeragenesis and directed evolution’), folding-function-fitness studies (Section ‘Three *f* determinants in TIM-barrel evolution: *f*olding, *f*unction, and *f*itness’), and protein design approaches (Section ‘Learning from nature towards protein design’). We illustrate how these studies pave the way to a detailed description of existing structure-folding-function-fitness relationships and also boost the design of new proteins with novel molecular properties.

## Current views of evolutionary units

Look at any protein and you are bound to find pieces that appear to have been reused either in different proteins or as the modules in a repeat protein. Clearly, reuse of sequences is ubiquitous within the natural fold space as was suggested already early on [1,2]. For protein scientists this beckons the question: how many of these pieces are there and what makes them so successful?

The structural annotation of proteins typically includes consulting at least one of the major databases SCOP, CATH or ECOD [3, 4, 5] to append additional information on evolutionary relationships. Molecular evolution studies have shown that different forces and mechanisms such as mutations, duplications, recombinations, deletions, and circular permutations drive the diversification of the protein-based world [6,7]. These mechanisms also hold true for events in the subdomain regime.

**Figure 2:**
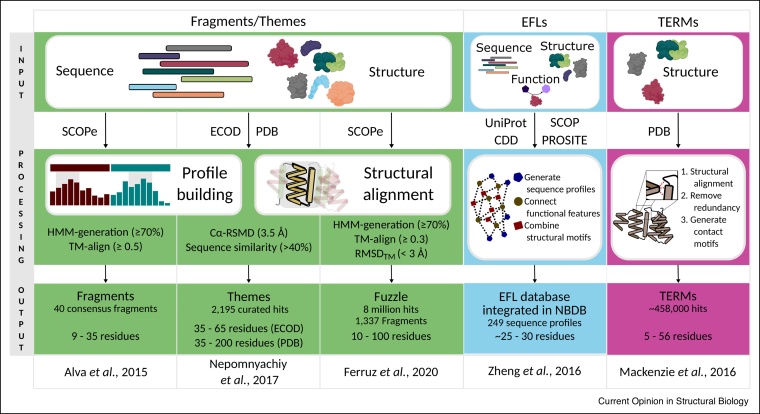
Current subdomain classification approaches. Shown is the generation of available subdomain databases including the different input, data processing, and final output. While Fragment/Themes are continuous sequences and are defined by HMM-profile comparisons and structural alignments, TERMs are non-continuous and focus on contact maps for classification. In contrast, EFLs combine information from structure, sequence and function, but are limited by existing annotation of functional sites.

In recent years there have been several approaches to define subdomain units as distinguishable building blocks (Figure 2). For example, an evolutionary relationship between the TIM-barrel and flavodoxin-like folds based on a 40-residue fragment was identified by sequence searches [8]. In a large-scale approach, Alva *et al.* identified and defined the reuse of elements within all modern proteins [9]. They generated a vocabulary of 40 subdomain fragments of up to 38 residues, which occur within a great number of different folds. Subsequent efforts to expand on these initial fragments led to the description of *themes* – reused fragments of at least 35 residues [10^••^]. A *theme* is defined whenever a sensitive sequence search using HHsearch suggests remote homology.

Along the same lines, Ferruz *et al.* expanded the fragment universe applying a set of filters to ensure the fragments are related, but not restricting their length [11^••^]. This generated a dataset of over eight million hits, which are summarized in the *Fuzzle* database (https://fuzzle.uni-bayreuth.de). When visualizing the dataset in a network representation a major component is observed that includes many hits between folds thought to be ancestral reinforcing earlier observations on different datasets [12,13]. This might hint not only to a common evolutionary history, but also to the existence of a favorable set of rules for protein folding, function, and fitness.

Another description by Berezovsky defines *elementary functional loops* (EFLs) [14]. These EFLs describe stretches of proteins with a specific sequence profile thought to be defined by the polymer nature of the polypeptide as reviewed recently [15]. Combining this with information on the conservation of structure and function provides indications, which elements might have proven successful in a primordial peptide-stage of evolution. This concept has been employed for example in the *nucleotide binding database* (NBDB), which contains EFLs involved in binding nucleotide-containing ligands [16]. Phosphate binding signatures obtained by this database were applied in the design of a P-loop protein testing the role of polymer physics in the emergence of basic units of proteins [17].

A fourth view that does not necessarily focus on the evolutionary aspect but rather on protein fold space are the *tertiary structural motifs* (TERMs) [18]. TERMs are 5–56 residue-long, discontinuous structural entities that are generated solely by comparing their environment. While TERMs focus primarily on conserved structural environments, a comparison of motifs generated by simulated evolution on TERMs and those of their natural counterparts showed that TERMs were able to accurately describe nature-like sequence variation.

These examples of either using structural information alone or sensitive in-depth sequence analysis or a combination thereof clearly hint to one thing: there is a subset of successful ancestral sequences that are to this day propagated to many modern folds.

## Evolutionary events: fragments and natural TIM-barrel proteins

**Figure 3:**
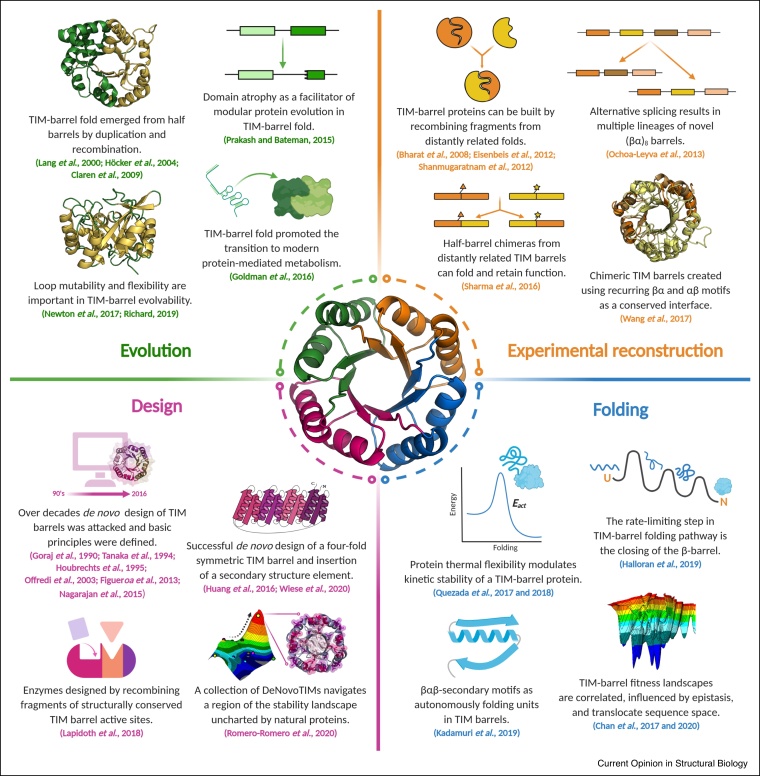
Summary of recent central studies that interconnect the evolution, its experimental reconstruction, folding, and design of TIM-barrel proteins as discussed in this review.

The previous section showed that, even after a considerable timespan, we can detect evolutionary relationships in modern proteins. Can we decode the underlying mechanisms of conservation of subdomain fragments in natural proteins? This general question has been explored by analyzing the evolution of different protein folds, for which the TIM barrel is a model system (Figure 3). This fold is regarded to be one of the oldest and encompasses a wide variety of known protein functions [19, 20, 21]. Its canonical fold consists of a central eight-stranded, parallel β-barrel surrounded by eight α-helices forming the eponymous (βα)_8_-barrel structure. It has previously been shown that subdomain parts of the TIM-barrel fold present an excellent model to probe the role of subdomain events, but also explore its evolution [22].

In a recent endeavor, Kadamuri *et al.* theorized that a set of βαβ sequences exists within the TIM-barrel fold-space, which would be autonomously folding units [23]. While there are not yet any reports of natural βαβ motifs folding in isolation, investigating the subdomain folding regime in TIM barrels might reveal crucial steps to improve the creation of novel proteins and help elucidate the evolution of protein domains themselves.

A study by Michalska *et al.* on the structural flexibility of naturally occurring TIM barrels reported a 3D-domain swap of an (αβ)_2_ element within a tryptophan synthase structure [24]. A similar event has recently been observed in a crystal structure of the archaeal chemotaxis protein CheY [25]. An analysis of alternative splicing events of (βα)_8_ barrels within the human genome also showed a considerable fraction expressing only as subdomains, and are thought to assemble to a complete barrel with their complementary partners [26]. These observations hint at a flexible subdomain composition within α/β proteins. This concept has been experimentally explored as will be discussed further in Section ‘Recreating evolutionary events in the lab: chimeragenesis and directed evolution’.

When Prakash and Bateman analyzed the variation of TIM-barrel domain boundaries, they found what they propose to be *domain atrophy* [27]. This rare event is characterized by a loss of core secondary structure features that is potentially detrimental to domain stability. While it is still not clear why such events are evolutionary fixed, a possible rescue of stability appears to be the formation of protein-protein interactions, for example, in homodimers.

All these examples of subdomain evolutionary events in the TIM-barrel fold point to one thing: there is a propensity of some proteins to swap subdomain elements. To really gauge if this subdomain recombination played — or still plays — an important role in the diversification of proteins, more protein folds need to be examined. Understanding the common principles that govern this process could help improve our knowledge of protein stability, folding, function and evolution.

## Recreating evolutionary events in the lab: chimeragenesis and directed evolution

The enormous diversity of protein structures and functions can be interpreted as the result of a massive *experiment* that has been carried out by Nature in a sustained way for millions of years, whose results are observed in the broad number of protein sequences and structures. In the previous section, we discussed that diverse evolutionary events in natural proteins allow the expansion of the protein fold space. Now, we focus on how some of these evolutionary events can be recreated in the laboratory through chimeragenesis and directed evolution. Both approaches offer a good alternative to test evolutionary and thermodynamic hypotheses and also to generate novel proteins (Figure 3).

Newton *et al.* explored the evolution of the TIM-barrel enzyme HisA using directed evolution techniques [28^••^]. They follow up on the innovation-amplification-divergence model previously proposed as an explanation of how gene duplication leads to proteins with new functions [29]. They show how beneficial substitutions selected during real-time evolution can result in manifold changes in enzyme function and bacterial fitness. The results emphasize the importance of loop mutability and confirms the TIM barrel as an inherently evolvable protein scaffold.

The current evolutionary hypothesis about the emergence of the TIM-barrel fold is that it evolved from duplication and fusion events of a half barrel, that is, a (βα)_4,_ or even smaller units [30, 31, 32, 33]. This possible pathway has been tested computationally and experimentally by analyzing sequence, structural, and folding properties [32,34,35]. Following this idea, Sharma *et al.* engineered and characterized active and stable chimeric TIM barrels of two distantly related glycosyl hydrolases, demonstrating that half-barrel domains from different sources can assemble and adopt the pre-evolved function [36]. Likewise, Almeida *et al.* tested the idea that (βα)_4_ halves are self-contained evolutionary units, independent of their size and internal symmetry. They introduced mutations in the inter-half contacts of a β-glucosidase to obtain independent half barrels that unfold cooperatively [37]. Further, Wang *et al.* identified physicochemical properties from a set of non-redundant TIM-barrel proteins that strongly support the existence of recurring βα and αβ motifs in this fold [38]. In addition, using a conserved αβα element as a recombination site, they created a chimeric protein from two different TIM barrels, highlighting the potential of recurring motifs as naturally optimized interfaces to engineer well-folded chimeras.

Inspired by TIM-barrel modularity, Lapidoth *et al.* designed highly active and stable enzymes by creating fragments of structurally conserved sites of two unrelated TIM-barrel families and then assembled them to create a large set of combinatorial backbones [39^••^]. The reported computational approach mimics natural evolutionary processes such as recombinations, insertions, deletions, and mutations, but it is more radical than these individual events since all of them are applied simultaneously to modify the protein fitness. As will be discussed in the last section (Learning from nature towards protein design), this method could be extended to create new biocatalysts by combining more distantly related families.

Apart from recombination events within a protein fold, recombination of heterologous structural motifs of unrelated folds is possible. Although difficult to detect in Nature, the idea can be tested in the laboratory and might be used to design proteins with novel biophysical properties [21]. In this context, ElGamacy *et al.* engineered an asymmetric dRP lyase fold fusing two heterologous and unrelated supersecondary structures. After interface optimization the approach generated a stable chimera with high precision to the original design [40^•^].

Similarly, we have used chimeragenesis in the past to elucidate evolutionary relationships of several α/β folds and design new proteins. Chimeras built combining parts of the flavodoxin-like proteins CheY or NarL with a piece of the TIM barrel HisF demonstrate that (βα)_8_-barrel proteins can be constructed by recombining a large repertoire of natural protein fragments from distantly related folds [8,41, 42, 43]. This interchangeability offers a great opportunity to retrace early evolutionary steps. Following up on this, Toledo-Patiño *et al.* found sequence-based evidence that the singleton HemD-like fold emerged from the flavodoxin-like fold [44^•^]. To test the hypothesized path, consisting of insert-assisted segment swap, gene duplication, and fusion, these evolutionary events were experimentally reverted, yielding well-folded and stable proteins. The results strongly support the emergence of the HemD-like fold from flavodoxin-like proteins and highlight the importance of duplication and fusion as evolutionary events that allow the creation of complex proteins. These experimental reconstructions of possible evolutionary events fit well with the bioinformatic studies on protein fragments as discussed in section ‘Current views of evolutionary units’. Databases such as *Fuzzle* [11^••^] provide many starting points for similar evolutionary explorations and open new ways to use already existing sequences in protein design. Fragments identified in *Fuzzle* can be used directly in the tool *Protlego* (https://hoecker-lab.github.io/protlego/) for automated chimera design and analysis [45].

## Three *f* determinants in TIM-barrel evolution: *f*olding, *f*unction, and *f*itness

The evolutionary study of biophysical determinants is useful to evaluate the role evolution has on the physical properties of proteins and informs us on how changes in the amino acid sequence shaped function in a specific fold [46]. In this section, we focus on recent advances to understand the biophysical basis underlying the success of the TIM-barrel fold as one of the most robust and versatile scaffolds.

The TIM-barrel fold provides a good architecture to explore how folding mechanisms have been conserved or diverged during evolution (Figure 3). In this context, Halloran *et al.* analyzed on a molecular level the earliest events in the folding of a TIM-barrel protein [47^••^]. Experimental and computational approaches revealed that the kinetic intermediate commonly observed in TIM barrels is dominated by a native-like structure in the central region of the sequence. They determined the rate-limiting step in the folding pathway to be the frustration encountered by the competition between the N-terminus or C-terminus to close the internal β-barrel. Also analyzing TIM-barrel proteins, Romero-Romero *et al.* studied and compared the folding pathway of eukaryotic homologous triosephosphate isomerases. Structural and biophysical analysis suggested that interfacial water molecules and water-mediated interactions could modulate the number of equilibrium intermediates, and therefore, the folding pathway in this enzyme family [48].

TIM-barrel proteins are notable for their diversity in catalytic activities. The broad presence of this topology in different enzymes has led to the assumption that the TIM-barrel fold played a central role in early evolution of catalysis. In a bioinformatic study, Goldman *et al.* showed by comparing the functional diversity of different protein folds that TIM-barrel proteins use the broadest range of enzymatic cofactors, including some putatively ancient cofactors [49^•^]. This supports the idea that the TIM barrel represented an ideal scaffold to facilitate the transition from ribozymes, peptides, and abiotic catalysts to modern protein-mediated metabolism.

Likewise, in terms of protein flexibility and enzymatic catalysis, Richard recently discussed why the selection and optimization of protein folds with multiple flexible loops, such as the TIM-barrel topology, is favored during enzyme evolution [50^•^]. He proposes that in TIM barrels the exploration of many different conformations during loop movement provides a potential starting point for the evolution of a new enzyme activity and allows the conformational changes needed in floppy enzymes. Also related with protein flexibility, but in the context of stability and evolution, Quezada *et al.* analyzed the molecular basis of the kinetic stability differences of two related triosephosphate isomerases and engineered new functional TIM-barrel enzymes with fine-tuned stabilities [51,52^•^]. They found a correlation between thermal flexibility and kinetic stability, suggesting how evolution has reached a balance between function and stability in cell-relevant timescales.

The evolution of protein folding, function, and fitness can be seen as a walk through sequence space, in the same way as was described 50 years ago by evolutionary biologist John Maynard Smith in his seminal work about natural selection and the concept of protein space [53]. Generally, each of these steps can be evaluated in terms of protein fitness, a measure of the effect that a property produces on the overall fitness of an organism. Following this logic, in two subsequent works the Matthews lab performed a quantitative description of the fitness landscape of distant orthologous TIM-barrel proteins to understand their evolutionary dynamics [54^••^,^55^]. They detected that the fitness landscapes are correlated and influenced by long-range epistatic interactions, and that these landscapes can be translocated in sequence space as a result of TIM-barrel fold plasticity.

The three *f* determinants in evolution discussed in this section have also been analyzed in other protein folds. Examples from the last years include discussions between the Makhatadze and Sanchez-Ruiz labs about the evolutionary validity of the minimal frustration hypothesis through the experimental characterization of ancestrally reconstructed proteins and extant homologous members of the thioredoxin family [56, 57, 58]. Also involving α/β proteins, Kukic *et al.* explored how the folding rates of Procarboxypeptidase A2 can be modulated during evolution by modification of the so-called nucleation-condensation mechanism [59]. Moreover, the Marqusee lab has made a substantial effort to understand how evolutionary pressures modify folding landscapes and tune kinetic and thermodynamic stability by characterizing one of the oldest protein folds, the RNase H-like superfamily [60, 61, 62, 63]. Other interesting works are the analysis of the influence of folding energies on the fitness of β-lactamases [64], the study of protein folding and fitness landscapes of amidases [65], the analysis of cotranslational folding and fitness of an integral membrane protein [66], and the evolutionary history of myoglobins [67]. The information obtained both on TIM barrels and other folds has revealed unanticipated details in protein molecular evolution thereby increasing our understanding of sequence-folding-fitness relationships, which has also relevant implications for protein design.

## Learning from nature towards protein design

In the previous sections we discussed the evolution of protein folds from smaller units and provided examples recreating such evolutionary events with respect to folding, function, and fitness. Same as protein engineering has been used to test evolutionary hypotheses, the gained knowledge can also be used to design new proteins. Initial protein design strategies were mostly based on parametrization of well understood folds or supersecondary structures. But in the last decades many powerful algorithms were developed to predict protein structures and design new proteins as has been recently reviewed [68].

One of the most widely used design software, namely Rosetta, uses 3-residue and 9-residue long fragments from known protein structures to sample the backbone in *ab initio* predictions [69,70]. Those fragments are a lot smaller than the previously described evolutionary units [9,10^••^,11^••^,^14^], however, they still can carry information about possible conformations. Additionally, some algorithms use evolutionary mechanisms as inspiration. The SEWING algorithm for instance incorporates current understanding of protein evolution, the emergence of proteins by recombination and duplication of smaller fragments: sets of structures meeting predefined requirements are generated by recombination of small structural motifs [71]. The more recently developed program dTERMen uses the previously described TERMs by matching them to the target design and thereby determines sequence preferences [72]. Also, the approach from Lapidoth *et al.* mentioned previously is inspired by Nature and mimics evolution during the design process [39^••^]. The fully automated method combines recombination, insertion, deletion, and mutation events in a non-sequential manner. Initially a predefined set of structures is partitioned and then assembled to combinatorial backbones, which are finally applied to a complete sequence redesign. During this process conserved sites and residues necessary for catalysis or folding can be excluded from the design. In contrast to other enzyme design approaches it has the advantage that no transition state has to be modelled which is computationally expensive. This method was applied to homologous TIM barrels but could possibly be extended to more distantly related proteins, thereby creating new biocatalysts. While this approach, that is based on existing structures, can diversify enzyme function, it will not create proteins from scratch.

The complete *de novo* design of proteins is a task that has been explored and progressed increasingly in recent years fueled by technical advances in structure determination, modelling, and computation. An increase in *de novo* designed proteins could be further observed after Koga *et al.* defined rules for the design of idealized protein topologies as recently reviewed [73]. The value of these design rules, that relate foldability of a tertiary structure to the connection between secondary structure elements [74], in combination with improvements in design algorithms can be traced in the design progression of *de novo* TIM barrels.

Several attempts were made to design a symmetric TIM barrel from scratch to understand what makes this protein fold so successful (Figure 3). In the early 1990s, first symmetric designs were created using statistical information about barrel geometries and amino acid frequencies from few known TIM-barrel structures [75, 76, 77, 78, 79, 80]. However, those parameters were not sufficient to achieve designs with natural-like properties as all exhibited molten-globule like states. With an increasing number of TIM-barrel structures, geometric parameters were improved, and newly emerging algorithms were applied to sequence design and created all-atom models. In this way, the Martial lab was able to improve previous designs and create natural-like proteins [81,82]. Later, the solubility of one of those designs was improved by directed evolution and the three-dimensional structure was determined: it differed from the intended TIM barrel and resembled a Rossman-like fold [83]. Using the previously described rules for idealized topologies, Nagarajan *et al.* created four-fold symmetric TIM-barrel backbones [84]. Using folding simulations, they determined hydrogen bond networks and enrichment of polar residues in the pore as important features regarding the folding pathway. Those findings were applied during iterative sequence design and resulted in soluble proteins showing cooperative unfolding transitions, though structural studies indicated a molten globule.

In the meantime, Huang *et al.* also applied the rules from Koga *et al.* to design a four-fold symmetric TIM barrel [85^•^]. Their approach sampled backbones with different secondary structure lengths using predefined geometric restrictions followed by iterative sequence design enforcing sidechain-backbone hydrogen bonds. A circular-permutated variant, sTIM11, was soluble expressed and the design was validated by solving its three-dimensional structure. Further analysis revealed a significantly lower conformational stability compared to natural TIM barrels. In a modular approach, a collection of stabilized variants (DeNovoTIMs) was designed by improving hydrophobic packing [86^••^]. Structural and folding analysis showed that epistatic effects allow navigating an unexplored region of the stability landscape of natural proteins. One of these DeNovoTIMs was already used in a successful recombination with a *de novo* designed ferredoxin protein and engineered to bind lanthanide [87]. In another recent study, Wiese *et al.* extended sTIM11 by successfully incorporating a rationally designed small α-helix into a βα loop [88]. These works are first steps towards diversifying and ultimately functionalizing *de novo* TIM barrels.

The progression in the design of a TIM barrel reflects nicely the improvements of protein design in the last 30 years. Throughout all design approaches, a symmetric topology was targeted as despite rapidly increasing computational resources the modelling of large proteins is still time-consuming. Further, this process shows how important it is to understand a protein fold in detail and to know which interactions are essential for its stabilization. In this context, it would be interesting to analyze the design from Figueroa *et al.* [82] in detail and determine why this design acquired a different fold than intended [83]. Such analysis is important to improve our understanding and find deficiencies in current protein design strategies.

Additionally, protein design opens a door not only to increase and test our knowledge about folding, function, and fitness, but also to compare the properties of *de novo* proteins with naturally occurring ones. In this way, studies have shown that *de novo* proteins exhibit more complex folding pathways than natural proteins, as indicated for one of the first *de novo* designed proteins Top7, a βα protein [89]. This differs from natural small proteins which show high cooperativity in folding and a smooth free energy surface. In addition, the study of another small *de novo* protein Di-III_14, an IF3-like protein, revealed a more complex folding pathway than initially assumed [90^••^]. In-depth mutational and folding analysis revealed that electrostatic and hydrophobic networks affect the energy surface of this protein. Based on those findings, it was proposed to limit the number of charged amino acids, avoid charge segregation, and use a more diverse set of nonpolar side chains in future protein designs. Overall, these studies demonstrate that as we expand our exploration into sequence space by designing *de novo* proteins, we also expand our understanding of the molecular and physicochemical determinants that shaped and still modulate the protein-based world.

## Conclusion and outlook

The study of protein evolution requires the integrated analysis of protein structure and stability, as well as folding, function, and fitness of proteins. There is clear evidence that modern diverse protein folds evolved via reuse of smaller units, which have been identified and described in recent years. Evolution of protein folds from smaller units via duplication has long been described, but also recombination is explored increasingly as an important mechanism. Understanding how protein diversity could emerge via these mechanisms is essential to learn how stable and functional proteins evolved and might be designed.

The ubiquitous TIM-barrel fold has been used in several studies to investigate its evolution, folding, and design. Explorations of the fold’s evolutionary history and experiments recreating evolutionary events have revealed how recombination of recurring fragments can lead to new proteins and enzymes. These studies go hand in hand with detailed analyses of protein folding and determination of fitness landscapes of TIM barrels. Moreover, this knowledge has already been applied to the design of *de novo* TIM barrels illustrating how the connection between evolution, folding, and design closes to a cycle and how analysis of designed proteins can help us understand the biophysical properties of proteins even better. Altogether, these recent studies have significantly increased our understanding of the evolution of sequence-structure-function relationships, enabling us to access new protein space through design.

## Conflict of interest statement

Nothing declared.

## References and recommended reading

Papers of particular interest, published within the period of review, have been highlighted as:

• of special interest
•• of outstanding interest
